# Using a medium-throughput comet assay to evaluate the global DNA methylation status of single cells

**DOI:** 10.3389/fgene.2014.00215

**Published:** 2014-07-07

**Authors:** Angélique Lewies, Etresia Van Dyk, Johannes F. Wentzel, Pieter J. Pretorius

**Affiliations:** ^1^Division for Biochemistry, School of Physical and Chemical Sciences, North-West UniversityPotchefstroom, South Africa; ^2^Centre of Excellence for Pharmaceutical Sciences, North-West UniversityPotchefstroom, South Africa

**Keywords:** medium-throughput comet assay, global DNA methylation, 5-Aza-dcR, single cells, cytosine extension assay (CEA), isoschizomeric restriction endonuclease

## Abstract

The comet assay is a simple and cost effective technique, commonly used to analyze and quantify DNA damage in individual cells. The versatility of the comet assay allows introduction of various modifications to the basic technique. The difference in the methylation sensitivity of the isoschizomeric restriction enzymes HpaII and MspI are used to demonstrate the ability of the comet assay to measure the global DNA methylation level of individual cells when using cell cultures. In the experiments described here, a medium-throughput comet assay and methylation sensitive comet assay are combined to produce a methylation sensitive medium-throughput comet assay to measure changes in the global DNA methylation pattern in individual cells under various growth conditions.

## INTRODUCTION

The comet assay has a long history of being used to assess the effects of various endogenous and exogenous substances on DNA damage and repair. Since, Ostling and Johanson (1984) showed that DNA from ϒ-irradiated cells migrate toward the anode due to the relaxation of the DNA supercoils the comet assay has been modified numerous times. These modifications range from altering the pH of the electrophoresis buffer (Calini et al., 2002), to exposing cells to various chemicals to assess the DNA repair capacity, to treatment of nucleoids with restriction enzymes (Andersson and Hellman, 2005) and even protein extracts to assess the effect of a given substance on DNA repair (Collins et al., 2001; van Dyk et al., 2010). Together with the still widely used standard comet assay, as described by Singh et al. (1988), the variety of modifications made to the comet assay perfectly showcase the adaptability and applicability of this technique.

The comet assay is an affordable and flexible method which can be easily adapted for the measurement of global DNA methylation. DNA methylation is not only important for maintaining genome stability but also plays an important role in gene regulation (Bird, 2002; Nag and Smerdon, 2009). DNA methylation is an epigenetic event which involves the chemical modification of DNA wherein the DNA sequence is not changed. In mammalian cells DNA methylation occurs at the cytosine residue of the CpG dinucleotide pair following each cycle of DNA replication and involves the addition of a methyl group at the carbon-5 position of cytosine through the action of DNA methyltransferases (DNMTs; Turker and Bestor, 1997; Espada and Esteller, 2010; Tost, 2010). DNA methylation patterns can be established on a global or gene-specific level in accordance with regulatory needs (Bird, 2002). The majority of CpGs in the genome are methylated, with the exception of CpG-islands which tend to remain hypomethylated in adult cells except on the inactivated X chromosome (French et al., 2009; Espada and Esteller, 2010). These CpG islands are characterized by relatively high CpG density. If the epigenetic processes are not correctly regulated, it may lead to changes in DNA methylation and histone modification patterns that disrupt important cellular processes, including gene expression, DNA repair and tumor suppression (Walsh and Xu, 2006; Li et al., 2007; Brooks et al., 2010; Tost, 2010).

The adaption of the comet assay to measure global methylation relies on the isoschizomeric properties of the two restriction enzymes: MspI and HpaII. These two isoschizomeric restriction enzymes recognize the same tetranucleotide sequence (5′-CCGG-3′) but display differential sensitivity to DNA methylation. HpaII is inactive when any of the two cytosines is methylated, but it digests the hemimethylated 5′-CCGG-3′ at a lower rate compared with the unmethylated sequences. On the other hand, MspI digests 5′-CmCGG -3′but not 5′-mCCGG-3′. These enzyme properties have been employed in other established techniques, such as the cytosine extension assay (CEA) and the luminometric assay (LUMA) for the measurement of global DNA methylation (Pogribny et al., 1999; Karimi et al., 2006). This difference is exploited to assess the global DNA methylation.

Some of the challenges and limitations of the methylation sensitive comet assay as previously reported (Wentzel and Pretorius, 2012) include, limited sample throughput, insufficient enzyme digestion of nucleoids and drying of agarose before enzyme digestion is complete. These challenges and limitations are; however, not unique to the methylation sensitive comet assay but are encountered in other adaptions of the comet assay as well. To address some of these limitations of the comet assay, a medium and high-throughput comet assay was developed (Stang and Witte, 2009; Azqueta et al., 2013; Gutzkow et al., 2013). Here we now describe combining a medium-throughput comet assay and a low-throughput methylation sensitive comet assay, to produce a methylation sensitive medium-throughput comet assay. This can then be used to assess the global DNA methylation status of single cells.

## MATERIALS AND METHODS

### CULTURE CONDITIONS AND METABOLITE TREATMENT

HepG2 cells were cultured in Dulbecco’s modified essential medium (D-MEM; Hyclone) containing 10% foetal bovine serum (FBS; Lonza), 1% penicillin/streptomycin (Lonza), 1% 200 mM L-Glutamine (Lonza) and 1% non-essential amino acids (Lonza). Cells were cultured at 37^∘^C in a humidified atmosphere of 5% CO_2_. For metabolite treatment, cells were seeded in 1.9 cm^2^ wells (24 well plate; Nunc^TM^) and cultured until confluent. The cells were subsequently cultured in the presence of 0.01 mM 5-azacytidine (5-Aza-dcR; Sigma–Aldrich) for 24 h. Following treatment, cells were harvested using 1× trypsin (Lonza).

### CYTOSINE EXTENSION ASSAY

The CEA was performed according to the method described by (Wentzel et al., 2010). Genomic DNA was isolated from 5-Aza-dCR treated cells using the DNeasy (blood and tissue) kit (Qiagen). The isolated DNA was subsequently separately digested with the endonucleases MspI and HpaII (Fermentas). The restriction enzyme mixture consisted of 1 μl of 1× Tango buffer (per 5 U of enzyme), 500 ng/μl DNA, and 10 U of enzyme (MspI/HpaII) in a final volume of 20 μl. The enzyme reaction was performed at 37^∘^C for 1 h followed by heat inactivation (65^∘^C for 15 min). The CEA reaction mixture consisted of 5× Taq buffer, 25 mM MgCl_2_, 5 U of GoTaq enzyme (Promega), and 0.1 μl of [^3^H] deoxycytidine triphosphate (dCTP; GE Healthcare) in a final volume of 15 μl. Subsequently 5 μl of the digested DNA was added to the 15 μl of the CEA reaction mixture and incubated for 1 h at 56^∘^C for the cytosine incorporation. The samples were transferred to Whatman DE-81 ion exchange filters (Whatman) and washed three times with 1× phosphate-buffered saline (PBS). The filters were air-dried at room temperature overnight. Scintillation counting in 9 ml Ultima Gold^TM^ XR (Perkin Elmer®;) was performed in a liquid scintillation analyzer (Perkin Elmer®; and Quantasmart^TM^ versio 3.00.5 Tri-Carb^TM^ LSC software). Background counts were subtracted from enzyme-treated samples, and the results were expressed as relative [^3^H] dCTP incorporation/0.5 mg of DNA and presented as percentage change from control samples. All samples were counted twice, and the average was calculated with sigma = 2%. The values were expressed as disintegrations per minute (dpm). Experiments were performed in triplicate.

### LOW-THROUGHPUT METHYLATION SENSITIVE COMET ASSAY

Modifications were made to the alkaline comet assay to detect changes in the levels of DNA methylation in single cells (Wentzel et al., 2010), using the 1 gel/slide format. During the harvesting process, cells are exposed to trypsin which may negatively influence the integrity of cells. Harvested cells were incubated in the D-MEM (containing 10% FBS) for 1 h at 37^∘^C in an orbital shaker to recuperate from the trypsin harvesting process. An 50 μl aliquot of the cell sample was mixed with 100 μl (15–20 cells/μl) of 0.5% low-melting-point agarose (LMPA; Fermentas) followed by the application of 100 μl of this solution to a frosted glass slide that had been pre-coated with a thin layer of 1% high-melting-point agarose (HMPA; Sigma–Aldrich). The slides were left at room temperature for the LMPA to set. The slides were subsequently submerged in lysing solution (consisting of 5M sodium chloride (NaCl; Sigma–Aldrich), ethylenediaminetetraacetic acid (EDTA; Sigma–Aldrich), 10% dimethyl sulfoxide [DMSO; Merck) and 1% Triton X-100 (Merck)] at 4^∘^C for 16 h to prepare nucleoids. The methylation sensitive comet assay employs the isoschizomeric restriction enzymes HpaII and MspI (Fermentas). To ensure favorable conditions for enzyme digestion, the slides were soaked in restriction enzyme reaction buffer (10 mmol/L Tris–HCl (Sigma–Aldrich), 10 mmol/L NaCl, 1 mmol/L mercaptoethanol (Sigma–Aldrich), and 2 mmol/L EDTA) for 10 min. Each enzyme mixture was composed of 1.5 unit of MspI or HpaII, 10 μl of Tango buffer (Fermentas) and filled to 100 μl with molecular grade H_2_O. 100 μl of this enzyme mix was subsequently applied to each slide and covered with a glass cover slip. The slides are then placed in a damp plastic container lined with towel paper that was preheated to 37^∘^C. After 5 min of incubation the slides are covered with towel paper soaked in reaction buffer to keep the slides from drying out while incubating for another 20 min. After incubation and removal of the coverslips, the slides were put into the electrophoresis tank and covered with electrophoresis buffer (5 mol/L NaOH and 0.4 mol/L EDTA). Electrophoresis took place at 30 V and 300 mA (between 0.8 and 0.9 V/cm) for 45 min at 4^∘^C, after which a pH neutralization step was performed by soaking the slides in 0.4 M Tris–HCl buffer (pH 7.5) for 15 min. Finally, the nucleoids were stained with ethidium bromide (10 μg/ml) for 1 h at 4^∘^C and rinsed with distilled water. The comet images were captured with an Olympus IX70 fluorescence microscope (200× magnification) and scored using Comet IV computer software version 4.3.1 (Perceptive Instruments Ltd). At least 200 comets were randomly scored per slide and the percentage of DNA migrating from the comet head (tail intensity) was measured for each comet scored. Experiments were performed in triplicate with two independent repeats.

### MEDIUM-THROUGHPUT METHYLATION SENSITIVE COMET ASSAY

For the medium-throughput methylation sensitive comet assay, a 12-well gasket (Severin Biotech) was used for the preparation of the comet slides and perform enzyme digestion. For cellular repair, the harvested HepG2 cells were incubated in D-MEM nutrient medium (containing 10% FBS) at 37^∘^C in an orbital shaker for 1 h. Frosted glass sides were pre-coated with 300 μl, 1% high melting point agarose (HMPA) and left to dry at room temperature for at least 1 h. The precoated slide was then placed into the 12-well gasket. Following the repair phase, a 50 μl aliquot of the cell sample was mixed with 100 μl of 0.5% low melting point agarose (LMPA) maintained at 40^∘^C. A volume of 20 μl (∼15–20 cells/μl) of this mixture was cautiously applied to each well and the aluminium gasket was placed on ice for 5 min for the LMPA to set. The nucleoids were exposed by adding 150 μl of lysis solution directly to each well and incubated at 4^∘^C for 1 h. Following cell lysis, each well was washed with 1x PBS (Sigma–Aldrich) at least twice. Nucleoids were treated with *Fast Digest* versions of the restriction enzymes HpaII and MspI (Fermentas). Each enzyme mixture was composed of 5 μl of MspI or HpaII, 5 μl of FB enzyme buffer (Fermentas) and filled to 50 μl with molecular grade H_2_O. Then 50 μl of this enzyme mixture was applied to each well and sealed with the silicone cap. The 12-well gasket was incubated at 37^∘^C for 30 min. Alternatively, a 1.0–1.5 mM solution of proteinase K (Qiagen) can also be employed to unwind the nucleus prior to enzyme digestion. This step contributes to making restriction enzyme recognition sites more accessible for MspI and HpaII. After incubation, the frosted glass plate was removed from the gasket and placed in electrophoresis buffer at 4^∘^C. After 30 min, electrophoresis was performed at 30 V and 300 mA (between 0.8 and 0.9 V/cm) for 45 min at 4^∘^C. Electrophoresis was followed by a pH neutralization step by soaking the slides in 0.4 M TrisHCl buffer (pH 7.5) for 15 min. Finally the nucleoids were stained with ethidium bromide (10 μg/ml) for one hour at 4^∘^C and thoroughly rinsed with distilled water. The comet images were captured with an Olympus IX70 fluorescence microscope (200× magnification) and scored using the Comet IV computer software version 4.3.1 (Perceptive Instruments Ltd). At least 400 comets were randomly scored per sample (between 50 and 100 comets per well) and the percentage of DNA migrating from the comet head (tail intensity) was measured for each comet scored. No less than nine replicates of three independent experiments were performed for each sample.

### STATISTICAL ANALYSIS

Statistical analysis was done with Prism 5 (GraphPad). For the Medium-throughput methylation sensitive comet assay, at least nine replicates were performed per sample and a minimum of 400 comets per sample were used for statistical analysis. Outliers were removed using the modified Thompson Tau method (Cimbala, 2011). In order to determine the distribution properties of the percentage CpG methylation, the bootstrap method was employed. A bootstrap replication number of 10,000 were employed with a 95% confidence interval. Percentage CpG methylation was calculated using the ratio between the average percentage tail DNA of HpaII- and MspI-digested DNA, that is, [(100–HpaII∖MspI × 100) –control], where HpaII and MspI are the average percentage tail DNA of HpaII- and MspI-digested nucleoids, respectively.

## RESULTS

**FIGURE 1:**
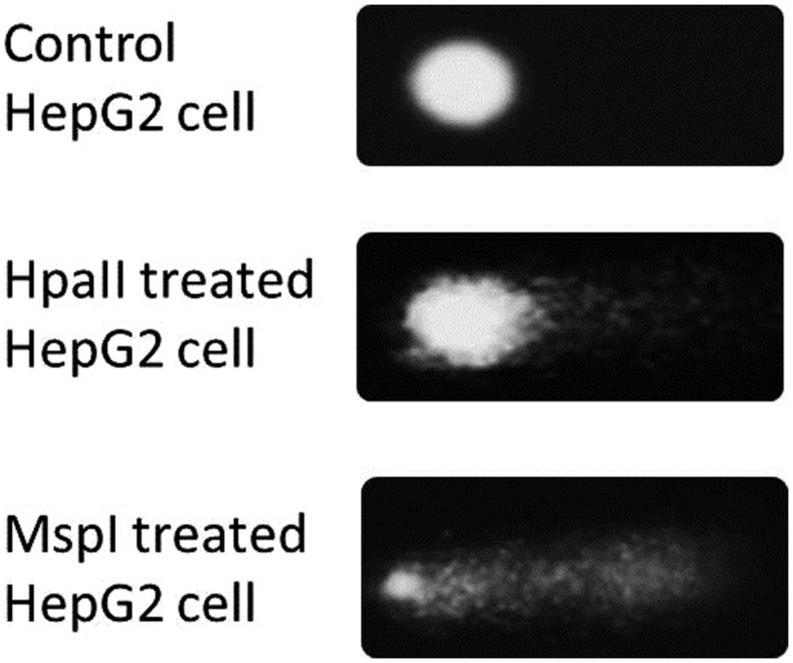
**Comets created by the treating nucleoids with the isoschizomeric enzymes MspI and HpaII**.

The methylation sensitive comet assay is based on the difference in sensitivity to DNA methylation of the two isoschizomeric restriction endonucleases HpaII and MspI. In theory, when these restriction enzymes are used in the comet assay, a higher level of methylation of the CpG dinucleotides should result in a larger difference in the amount of DNA in the comet tails of HpaII-digested nucleoids versus MspI-digested nucleoids. From **Figure 1** it is evident that the treatment of agarose-embedded nucleoids with MspI indeed resulted in markedly more comet tail DNA relative to the undigested control. Similarly, a smaller but still significant, increase in the tail DNA is observed following HpaII treatment.

**FIGURE 2:**
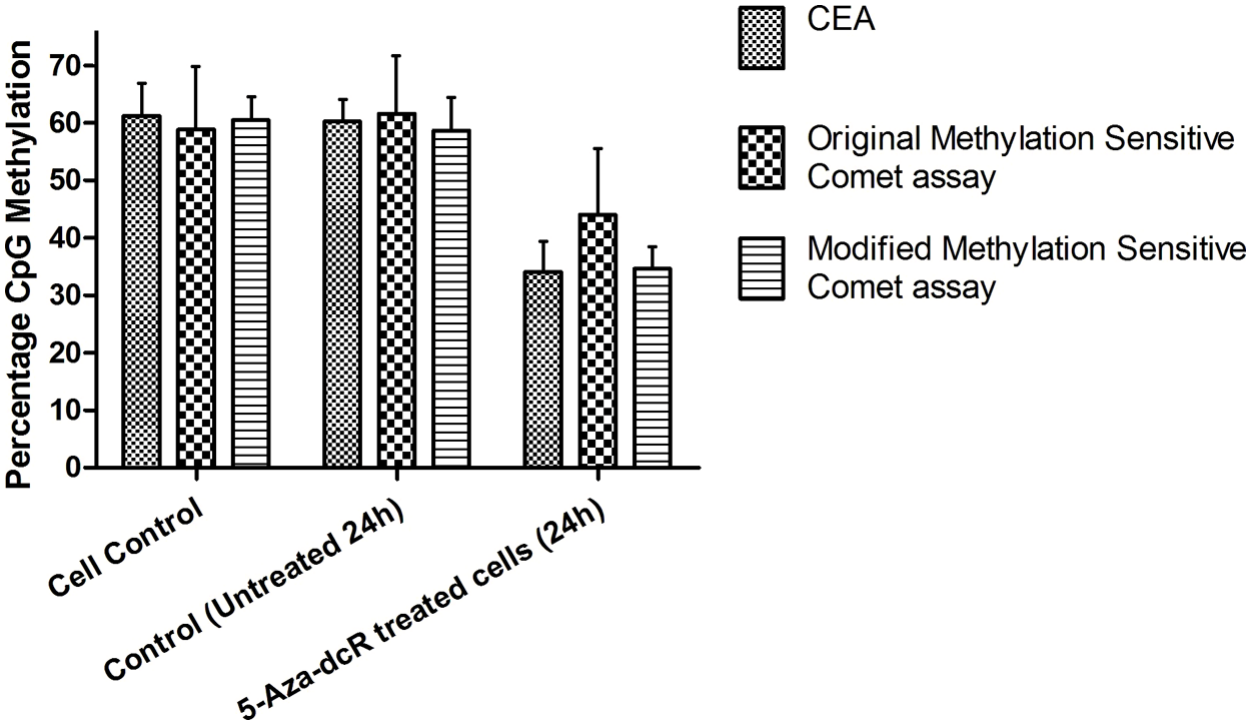
**DNA methylation of HepG2 cells treated with 5-Aza-dcR.** Comparison between global CpG methylation of cells in culture under normal conditions and treated with the demethylation agent 5-Aza-dcR using the two methylation sensitive comet assays and the CEA. All experiments were at least performed in triplicate with two independent repeats.

**FIGURE 3:**
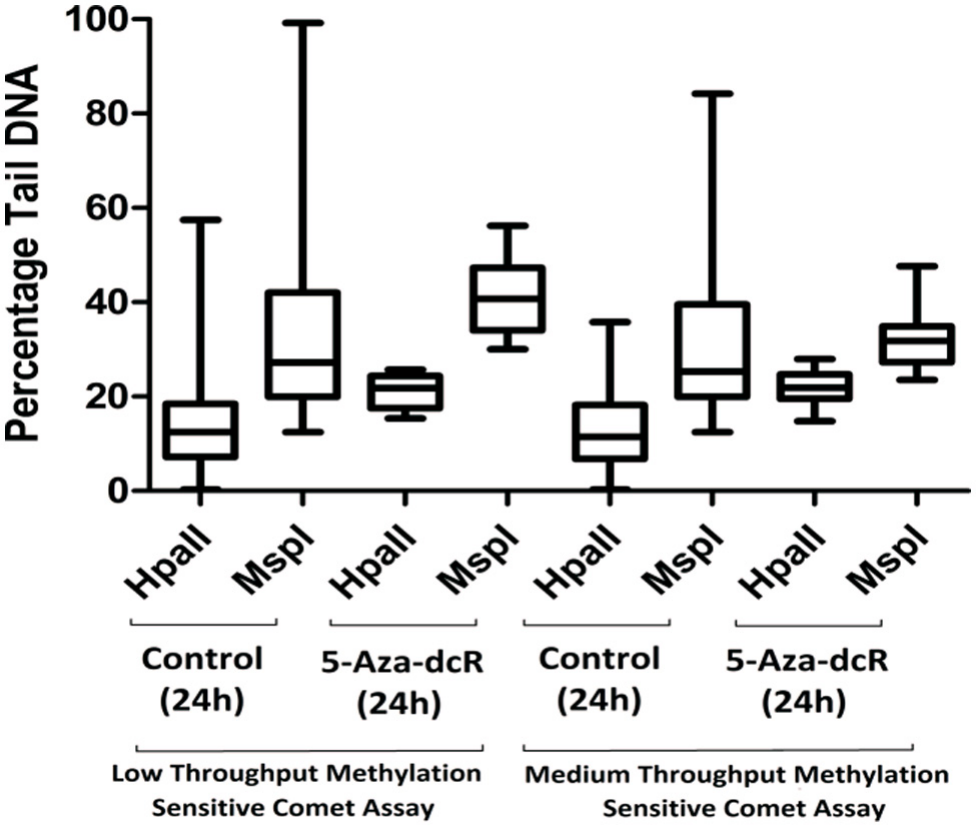
**A comparison of the percentage tail DNA in comets of cells cultured under normal conditions in comparison with cells treated with the demethylation agent 5-Aza-dcR as measured with the low- and medium-throughput methylation sensitive comet assays.** A bootstrap replication number of 10,000 were employed with a 95% confidence interval. All experiments were at least performed in triplicate with two independent repeats.

To improve the low-throughput methylation sensitive comet assay, a 12-well gasket was used for the preparation of the comet slides and enzyme digestion. The original low-throughput and modified medium-throughput comet assays were then compared. The results are expressed as percentage CpG methylation and are calculated using the ratio between the average percentage tail DNA of HpaII- and MspI-digested DNA. The results of the two methylation sensitive comet assays were validated using the CEA on DNA isolated from the remaining cells of the same batch used for the comet assay (**Figure 2**). The calculated percentage CpG methylation is 62.2 and 58.6% for untreated cells and 44.0 and 34.6% for 5-Aza-dcR-treated cells detected by the low-throughput and medium-throughput methylation sensitive comet assays, respectively. For the CEA data set, the percentage CpG methylation is 60.2% for untreated cells and 34.0% for 5-Aza-dcR-treated cells. A comparison of the distribution of the percentage CpG methylation of the low-throughput methylation sensitive comet assay in comparison to the medium-througput methylation sensitive comet assay is depicted in **Figure 3**. The area between the first- and third quartile for percentage CpG methylation is smaller in data generated with the medium-throughput methylation sensitive comet assay in contrast to the low-throughput method, in which percentage CpG methylation is more widely distributed.

## DISCUSSION

Although a variety of techniques are used to measure global DNA methylation patterns, most of these techniques are expensive and platform specific (Shen and Waterland, 2007; Lisanti et al., 2013). The comet assay is a cost-effective, sensitive, and simple technique, which is traditionally used for analyzing and quantifying DNA damage in individual cells (Fairbairn et al., 1995; Azqueta et al., 2011). Nowadays this method is regularly used in biomonitoring and mechanistic studies in a large range of *in vitro* and *in vivo* systems (Dusinska and Collins, 2008; Valverde and Rojas, 2009; Cemeli and Anderson, 2011). The comet assay is also widely used for genotoxicity studies and determining DNA repair capacity and a variety of DNA lesions can be detected, including DNA double strand breaks (DSB) and single strand breaks (SSB), as well as alkali-labile sites (Fairbairn et al., 1995; Collins and Gaivao, 2007).

The use of specific restriction endonucleases with the comet assay expands the flexibility of the method. The comet assay can be modified through the use of lesion specific restriction endonucleases to detect specific base modifications as DNA SSB (Epe et al., 1993; Tice et al., 2000; Collins and Gaivao, 2007; Collins, 2009; Speit et al., 2009). In a similar way the comet assay can be modified to measure DNA methylation by using methylation sensitive restriction endonucleases. By doing this it is possible to simultaneously measure global as well as CpG island DNA methylation and DNA damage and repair in a variety of cells (Wentzel et al., 2010).

The use of methylation sensitive restriction endonucleases can modify the traditional alkaline comet assay to be methylation sensitive. Similar to the CEA that measures global DNA methylation (Pogribny et al., 1999), the methylation sensitive comet assay also employs the isoschizomeric restriction endonucleases HpaII and MspI. As previously mentioned, these enzymes recognize the same tetranucleotide sequence (5′-CCGG 3′) but display differential sensitivity to DNA methylation (**Figure 1**). Unmethylated DNA is digested by HpaII, however, when either of the two cytosines are methylated HpaII will not digest the DNA. When the DNA is hemimethylated, i.e., only one of the two complimentary strands are methylated, HpaII will digest the DNA, but at a slower rate than digestion of unmethylated DNA. Conversely, MspI will digest methylated DNA, but only 5′-CmCGG-3′ and not 5′-mCCGG-3′ (Tost and Gut, 2010).

Even though theoretically the percentage tail DNA following MspI treatment represents all of the 5′-CCGG-3′ sites in the DNA, it is important to note that when using MspI and HpaII only the methylated cytosines outside of CpG islands are quantified as these enzymes tend to mainly recognize sequences outside of CpG islands. Cytosines within these regions tend to be methylated whereas cytosines in the CpG islands tend to be unmethylated (Shen and Waterland, 2007). The global 5′-CCGG-3′ methylation can be calculated by the HpaII/MspI ratio. Compensation is made for DNA damage prior to enzyme treatment by subtracting the percentage tail DNA from the control samples.

In the current study the previously modified method (Wentzel et al., 2010) was further adapted by using *Fast Digest* versions of the HpaII and MspI restriction endonucleases and the 12 gels/slide format of the comet assay (Shaposhnikov et al., 2010). In short, HepG2 cells were exposed to the demethylating agent 5-Aza-dcR for 24 h as exposure to this demethylating agent causes a decrease in the percentage global DNA methylations. Results from the conventional 1 gel/slide format (low-throughput comet assay) was compared to the use of 12 gels/slide format (medium-throughput comet assay) using the *Fast Digest* versions of HpaII and MspI and validated with the established CEA (Pogribny et al., 1999).

A similar decrease in the percentage CpG methylation following 5-Aza-dcR treatment for the CEA and the medium-throughput methylation sensitive comet assay (26.2 and 24 %) and comparatively lower decrease in percentage CpG methylation for the low-throughput methylation sensitive comet assay following 5-Aza-dcR treatment was seen (**Figure 2**). **Figure 3** furthermore showed that the distribution of the data was also better for the medium-through put comet assay compared to the low-throughput comet assay.

The results for the medium-throughput methylation sensitive comet assay and the CEA following 5-Aza-dcR treatment are similar due to the fact that the enzyme digestion conditions are nearer to that recommended by the manufacturer and closer to the conditions used in the CEA. The enzyme digestions are performed in individual wells and a silicon cover is placed over the gasket forming a lid over each individual well during incubation. In the low-throughput method the enzyme/buffer mixture is spread over the entire gel, a glass cover slide is placed over the frosted glass slide and it is then incubated in a damp plastic container. In the later method the enzyme/buffer mixture tends to evaporate, which changes the enzymatic reaction conditions.

The use of the 12-gels/slide instead of the traditional 1 gel/slide not only upgraded the comet assay to a medium-throughput method, the 12-well gasket greatly improved the restriction enzyme digestion conditions and considerably reduced consumable use. The deployment the *Fast Digest* versions of the restriction enzymes HpaII and MspI, further also improved nucleoid digestion and reduced incubation time. This modified method also overcomes “edge-effects” as observed when the traditional frosted glass slides are used.

## CONCLUSION

The difference in methylation sensitivity of the isoschizomeric restriction endonucleases HpaII and MspI may be exploited to demonstrate the feasibility of using the comet assay to measure global DNA methylation level in individual cells. In the present study we showed that the comet assay can be modified to measure global DNA methylation in single cells in a medium-throughput manner. The use of the 12-well gasket to perform the enzyme digestions offers more ideal conditions for enzyme digestion and overcomes some of the limitations that are faced when restriction enzymes are used in conjunction with the comet assay, such as “edge-effects,” sub-optimal enzyme reaction conditions and gel drying.

The use of the comet assay over other methods such as CEA for the measurement of global DNA methylation offers the advantage that it is less expensive. Furthermore, DNA methylation is tissue specific (Pogribny et al., 1999) and this method can be used to measure the changes in the global DNA methylation pattern of a variety of cells under different physiological conditions on a single cell level.

The versatility of the comet assay is further expanded through the modifications made in this study, increasing the number of observations that can be made with a single experiment and reducing the amount of labor and inter-experimental variability.

## AUTHOR CONTRIBUTIONS

Angélique Lewies, Etresia Van Dyk, Johannes F. Wentzel, and Pieter J. Pretorius (principal investigator) designed the study. Angélique Lewies, Etresia Van Dyk, Johannes F. Wentzel performed the experiments, processed the data, and wrote the manuscript.

## Conflict of Interest Statement

The authors declare that the research was conducted in the absence of any commercial or financial relationships that could be construed as a potential conflict of interest.
